# Analysis of the regulatory mechanisms of prognostic immune factors in thyroid cancer

**DOI:** 10.3389/fonc.2022.1059591

**Published:** 2022-12-14

**Authors:** Yin Tian, Tao Xie, Xue Sun

**Affiliations:** ^1^Department of Pediatric Surgery, Jingzhou Central Hospital, Jingzhou Hospital Affiliated to Yangtze University, Jingzhou, Hubei, China; ^2^Department of Anesthesiology, Jingzhou Central Hospital, Jingzhou Hospital Affiliated to Yangtze University, Jingzhou, Hubei, China; ^3^Department of Clinical Nutrition, Sinopharm Dongfeng General Hospital, Hubei University of Medicine, Shiyan, Hubei, China

**Keywords:** thyroid cancer, immune gene, prognosis, miRNA, targeted combination

## Abstract

**Objective:**

To explore the regulatory mechanism of immune prognostic factors in thyroid cancer.

**Methods:**

Based on the TCGA database and GEO database, this study used bioinformatics methods to study the potential regulatory mechanism of thyroid cancer prognosis, analyzed the differentially expressed genes and differential miRNAs between thyroid cancer and normal paracancerous tissues by R software, and constructed lasso risk factors. The immune prognostic factors of thyroid cancer were obtained from the model, and the miRDB website was used to predict the possibility of differential miRNA target binding of the immune prognostic factors and correlation analysis was performed, and finally verified by cell experiments.

**Results:**

There were 1413 differentially expressed genes between thyroid cancer and normal paracancerous tissues, among which 21 immune-related genes were prognostic factors with significant differences in expression; lasso risk model obtained AKAP12, APOC1, TIMP3, ADAMTS9, ANK2, HTRA3, SYNDIG1 , ADAMTS5 and DACT1 were nine prognostic factors. A total of 58 differential miRNAs were found in thyroid cancer tissues and non-cancerous tissues. The possibility of differential miRNA targeting and binding of immune prognostic factors on the miRDB website and cell experiments was analyzed.

**Conclusions:**

The potential miRNA regulatory mechanism of immune prognostic factors in thyroid cancer has been explored.

## Introduction

Thyroid cancer is a common endocrine malignancy (1, 2). Although THCA has a low mortality rate and a relatively good prognosis, the existing therapeutic modalities for locally advanced or recurrent metastatic THCA are still ineffective in improving the prognosis of patients. Therefore, new therapeutic modalities targeting the molecular mechanisms of thyroid carcinogenesis and progression are still being explored.

Recent studies have revealed that normal cells must possess the intrinsic characteristics of tumor cells to develop into cancer cells; most importantly, tumor cells can evade antitumor immune responses and form tumor foci (3). Antitumor immunity is an essential mechanism for the host’s inhibition of tumorigenesis. However, tumor cells can evade the body’s immune attack, survive, and proliferate to form tumor foci (4, 5). Tumor cells can play a crucial role in disease development through different immune mechanisms. Immune-related genes have been shown to play a role in cancer development (6–8) and, in some studies, can also can predict the prognosis of cancer patients and as therapeutic targets (9–12). In addition to being an effective prognostic biomarker by constructing the immune prognostic features of cancer using bioinformatics, it is also a promising and novel therapeutic target. Wang et al. (13) found that an immune-related prognostic signature consisting of *SLC10A2, FGF2, CCL28, NDRG1, ESM1, UCN, UTS2*, and *TRDC* could effectively predict 3- and 5-year overall survival rates. Heterogeneous expression of immune-related genes can be used as a prognostic factor, but the regulatory mechanism for the heterogeneous expression of immune factors is unknown.

Micro ribonucleic acids **(**miRNAs) are conserved endogenous short-stranded noncoding ribonucleic acid (RNA) molecules consisting of 21-23 nucleotides. miRNAs have been shown to target multiple messenger RNAs (mRNAs) and are involved in almost every biological process (14, 15). In recent years, studies targeting the role of miRNAs in cancer based on miRNA expression profiles have increased annually. Many miRNAs are down- or up-regulated in human cancers and act as oncogenic or intra-cancer suppressors (16). In ovarian cancer, many miRNAs regulate the epithelial-mesenchymal transition program (17). At the level of molecular mechanisms, miR-125a and miR-125b can regulate immune cell development and function, thus acting as tumor suppressors or promoters (18).

In this study, a total of nine prognostic immune factors, *AKAP12, APOC1, TIMP3, ADAMTS9, ANK2, HTRA3, SYNDIG1, ADAMTS5*, and *DACT1*, were obtained by screening differentially-expressed genes and differential miRNAs using LASSO analysis and analyzing the differential miRNA. The target-binding relationship between differential miRNAs and prognostic immune factors was also analyzed. We validated them using correlation analysis to find the regulatory mechanism of immune prognostic factors in THCA.

## Materials and methods

### Data sources

The data sources for this study were the TCGA and GEO databases. Those databases included RNA sequencing (RNAseq) data and clinicopathological information from 510 THCA samples; mRNA data from 58 normal tissues adjacent to the cancer were obtained. The tissue from 29 THCA patients and five non-THCA patients was obtained from the GEO database GSE103996 dataset miRNA expression profile. Immune-related genes was got from the Immunology Database and Analysis Portal database (https://www.immport.org).

### Differential expression analysis

Differential expression genes(DEGs) and differentially-expressed miRNAs of THCA tissues and paraneoplastic tissues were analyzed differentially using the R package limma package, where differentially-expressed genes were screened at p < 0.05, |log2FC|>1, and differentially-expressed miRNAs were screened at p < 0.05, |log2FC|>1, and represented in volcano and heat maps using Venn diagrams to take the intersection of the screened DEGs with immune-related genes.

### Prognostic analysis

The transcriptional expression profile data and information of thyroid cancer patients obtained from the TCGA database were plotted using Kaplan–Meier curves with R software. P-values and hazard ratios with 95% confidence intervals (CI) were derived using a log-rank test and univariate Cox regression; the data were presented as forest plots.

### The prognostic signature model

The relationship between prognostic immune-related gene expression and overall survival (OS) was assessed using LASSO COX analysis. the prognostic risk prediction model for THCA was based on the LASSO risk score calculation formula. patients with THCA were divided into high-risk and low-risk groups. KM curves were plotted to compare OS in the high-risk and low-risk fractions. receiver operating characteristic survival analysis was performed using the R package SURVIVAL; decision curve analysis was performed using the risk model decision analysis (rmda) package. The relationship between risk score models and tumor immune infiltrating cells was also observed.

### Single gene expression analysis

RNAseq data in level 3 HTSeq-FPKM format or miRNAseq data in level 3 BCGSC miRNA Profiling from the TCGA THCA (thyroid cancer) project. The miRNAseq data in fragments per kilobase per million (FPKM) or reads per million mapped reads (RPM) format was log2-transformed; data significance was p < 0.05; *, p < 0.05; **, p < 0.01; ***, and p < 0.001.

### Target gene miRNA prediction and correlation analysis

Target gene miRNA prediction was performed using the microRNA target prediction database (miRDB) website (http://mirdb.org/). Transcriptional profiles and miRNA expression profile data were obtained from the TCGA database. Correlation plots between the two were implemented using the R ggstatsplot package. Spearman’s correlation analysis can perform correlations between quantitative variables, which were statistically significant with a p-value less than 0.05.

### Cell culture and RT-qPCR

RT-qPCR assays for human normal thyroid cell line Nthy-ori 3-1 and human THCA cell line ACT-1 were purchased from the American Type Culture Collection. All cell lines were cultured in RPMI-1640 containing 10% fetal bovine serum (FBS, Gibco), 1% penicillin/streptomycin, and maintained in a 37°C, 5% CO_2_ incubator. Total RNA was extracted from each group of cells using TRIzol reagent (Invitrogen, Carlsbad, CA) following the manufacturer’s guidelines. RNA was synthesized into cDNA using Primescript RT Reagent Kit (Takara, Dalian, China). cDNA amplification and quantification were performed on an Applied Biosystems 7500 instrument using SYBR Green mix (Takara). The relative expression of mRNA was calculated using the 2^-ΔΔCt^ method, and the experiment was repeated three times independently for each sample. miRNA was used as an internal reference for U6, and glyceraldehyde-3-phosphate dehydrogenase (GAPDH) was used as an internal reference for genes. The PCR primers are shown in **Table 1**.

**Table 1:** Sequence of primers.

Primer name	Sequences
ADAMTS5-F	GAACATCGACCAACTCTACTCCG
ADAMTS5-R	CAATGCCCACCGAACCATCT
ADAMTS9-F	TGGGTTTTCCAGTTTTCAG
ADAMTS9-R	GTTGATGCTAAAACGACCC
ANK2-F	ACCTGCGATACAGCTTGGAG
ANK2-R	AGAGTGTGAGACCTGTCGGA
AKAP12-F	CTGCCTTGGGAGTTTGCC
AKAP12-R	GGGTTACGCCTTCCCCAAG
APOC1-F	ACCCACTT AGAGTTGTGAGCCC
APOC1-R	CAGACCACCTTAGTCCCTTTCC
DACT1-F	AGATATCCCCTTGGCACCCT
DACT1-R	TTCAGTGAGAGTCCACCACA
HTRA3-F	TGACCAGTCCGCGGTACAAG
HTRA3-R	TTGGAGCTGGAGACCACGTG
TIMP3-F	CTCGAGCAAGGAGGAACTTGGGTG
TIMP3-R	GCGGCCGCAATACAGAAGTGTCT
SYNDIG1-F	CCTTGTCCCGGAGCCCA
SYNDIG1-R	ACAGACGTGGAGCACTGAAG
miR-15a-5p-F	CGCGTAGCAGCACATAATGG
miR-15a-5p-R	AGTGCAGGGTCCGAGGTATT
miR-30c-5p-F	GCGCGTGTAAACATCCTACACT
miR-30c-5p-R	AGTGCAGGGTCCGAGGTATT
miR-34b-5p-F	GGGTAGGCAGTGTCATTAGC
miR-34b-5p-R	AACAACCAACACAACCCAAC
GAPDH-F	GGAGCGAGATCCCTCCAAAAT
GAPDH-R	GGCTGTTGTCATACTTCTCATGG
U6-F	GCTTCGGCAGCACATATACTAAAAT
U6-R	CGCTTCACGAATTTGCGTGTCAT

### Statistical analysis

Cellular tests were performed using the IBM Statistical Package for the Social Sciences (SPSS) 24.0 and GraphPad Prism8.0.1 software to process the data. Normally distributed measures were expressed as (x ± s), and an independent samples t-test was used to compare the two parts. A one-way analysis of variance can comparison between multiple parts, and Tukey’s multiple comparisons test was used for *post hoc* tests. P was a two-sided test, and differences were statistically significant at p < 0.05.

## Results

### Immunoprognostic analysis of differentially significant genes in thyroid cancer

**Figure 1:**
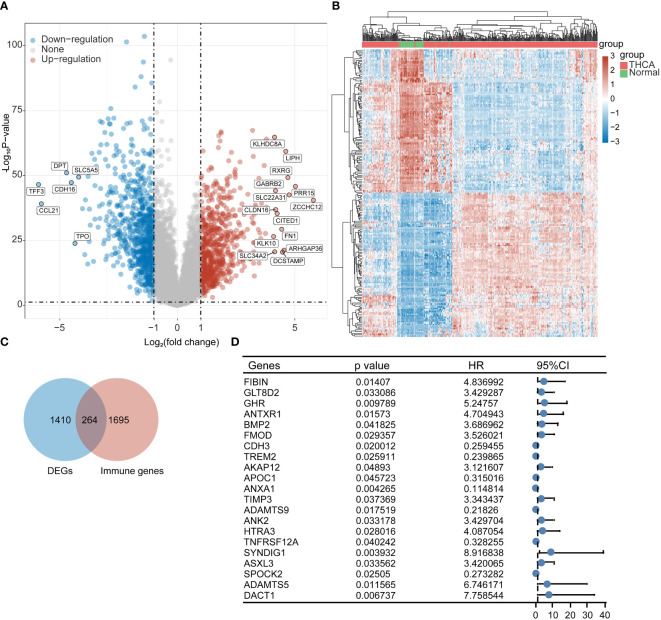
Screening of immune-related prognostic genes in DEGs. **(A)** Volcano plot, **(B)** Heat map, **(C)** Wayne plot, and **(D)** 21 differential genes with prognostic value.

We obtained transcriptomic data from 510 thyroid cancer patients and 58 paraneoplastic tissues from the TCGA database for differential gene analysis; a total of 676 up-regulated genes and 737 downregulated genes were obtained by |log2FC|>1 with p < 0.05. **Figure 1A** shows the volcano plot, and **Figure 1B** shows the heat map. We then obtained 264 identical genes by taking the intersection of the DEGs and immune gene sets (**Figure 1C**). We performed a prognostic analysis of these 264 genes based on individual gene expression using both one-way Cox and log-rank tests. We obtained a total of *FIBIN, LT8D2, GHR, ANTXR1, BMP2, FMOD, CDH3, TREM2, AKAP12, APOC1, ANXA1, TIMP3, ADAMTS9 ANK2, HTRA3, TNFRSF12A, SYNDIG1, ASXL3, SPOCK2, ADAMTS5*, and *DACT1* 21 prognostic genes with significantly different immune-related expression (**Figure 1D**).

### Immunogenic prognostic model construction

**Figure 2:**
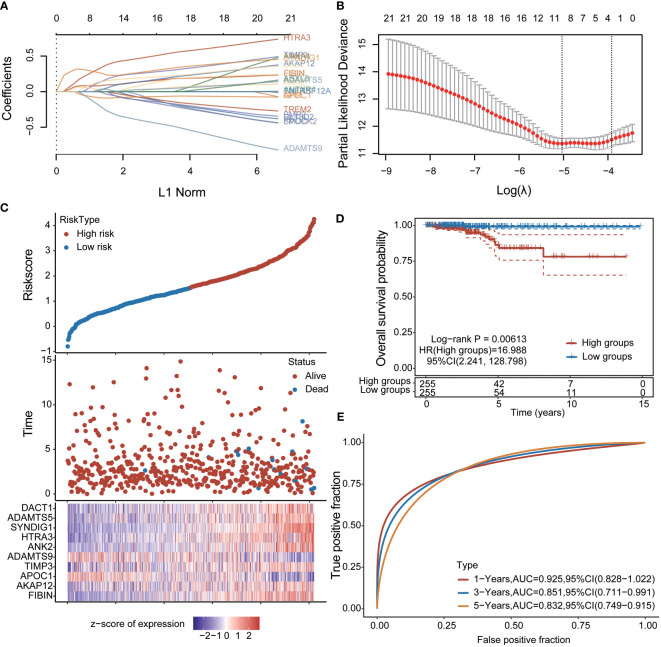
Construction of prognostic features of immune-related genes in thyroid cancer **(A, B)** distribution of LASSO coefficients of 21 pivot genes, the adjustment parameter λ.min= 0.0065 is obtained, and the vertical black dotted line in B defines the optimal λ value; **(C)** Thyroid cancer Risk score (Riskscore) distribution of patients, survival status and duration of thyroid cancer patients **(D)** KM survival curves of high and low-risk groups; **(E)**: ROC curves of 1 year, 3 years and 5 years.

To further explore the clinical value of significant differences in immune-related expression, prognostic features were established for 21 immune-related genes based on LASSO Cox analysis (**Figures 2A, B**). Using risk score Riskscore = (0.0819)*FIBIN+(0.0058)*AKAP12+(-0.0814)*APOC1+(0.1218)*TIMP3+(-0.1791)*ADAMTS9+(0.0335)*ANK2+(0.3115)*HTRA3+(0.2709)*SYNDIG1+(0.0237)*ADAMTS5+(0.1317)*DACT1, THCA patients were divided into high and low-risk groups (**Figure 2C**); Survival status is shown in **Figure 6D**. The figure shows the difference in survival between the high and low-risk parts (p = 0.00613). Prognostic survival was predicted for 1, 3, and 5 years; the model showed good sensitivity (**Figure 6E**). In summary, nine prognostic factors were obtained for *AKAP12, APOC1, TIMP3, ADAMTS9, ANK2, HTRA3, SYNDIG1, ADAMTS5*, and *DACT1*.

### Significant differences exist in miRNAs regulating the heterogeneous expression of prognostic factors

**Figure 3:**
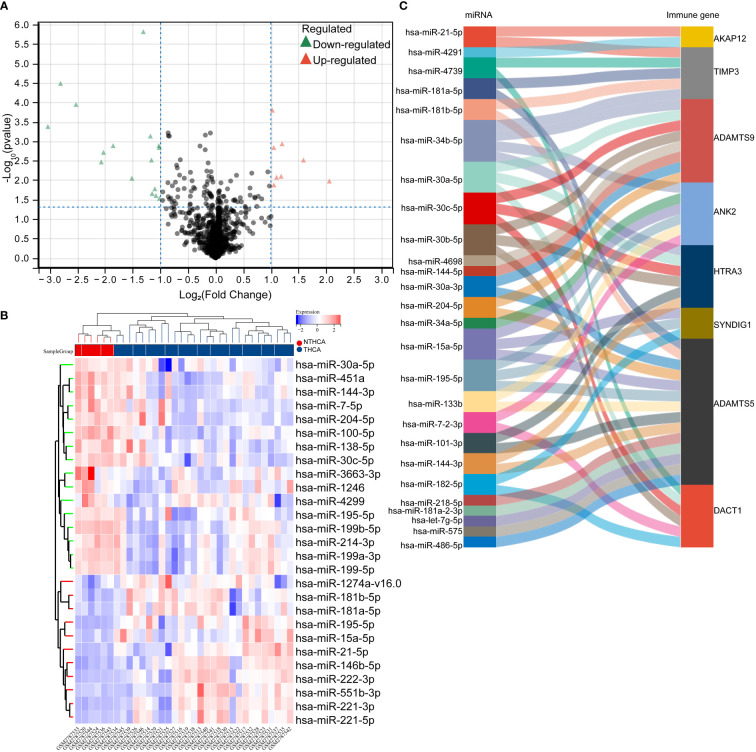
Targeted regulation of differentially significant miRNAs that regulate heterogeneous expression of prognostic factors. **(A, B)** volcano plot of differentially significantly expressed miRNAs in the GSE103996 dataset, heat map; **(C)** Sankey plot of miRNAs that target predicted binding to prognostic factors.

To understand the heterogeneous expression mechanism of immune prognostic factors in thyroid cancer and adjacent tissues, we introduced the miRNA transcriptome information of thyroid cancer and non-cancer tissues into the GSE103996 dataset of the GEO database, and obtained 58 differential miRNAs through analysis. Among them, 21 were up-regulated and 37 were down-regulated. The heat map is shown in **Figure 3B**. We then screened for miRNAs that bind to postimmune factors, and their targeting relationships are shown in the Sankey diagram in **Figure 3C**.

### Targeted binding miRNA expression to prognostic factors and prognosis

**Figure 4:**
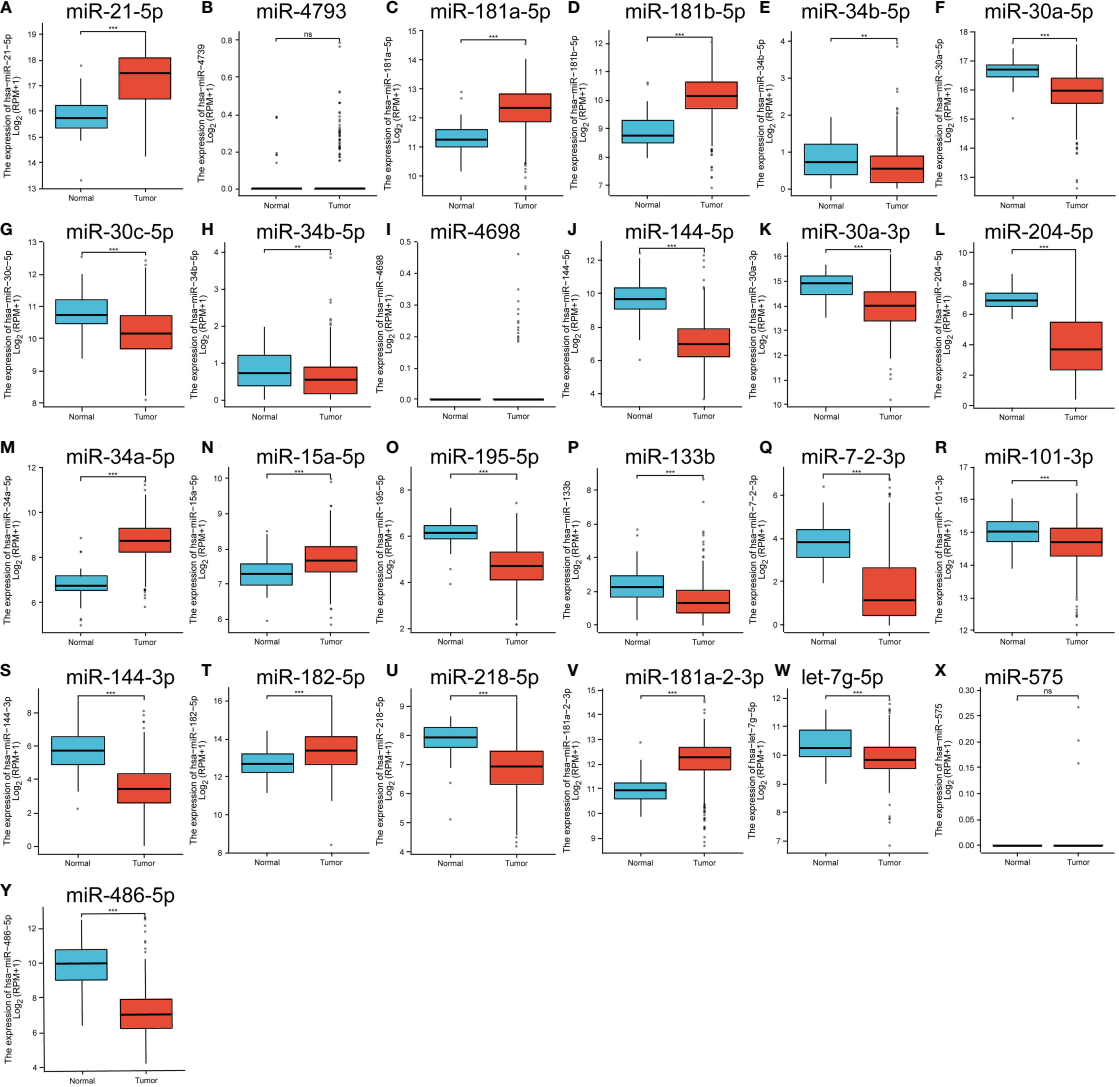
**(A–Y)** is the expression of significantly different miRNAs in the GEO database that have a targeted binding relationship with the prognostic factors in the TCGA database. ns means no statistical significance, ** means p<0.01, *** means p<0.00.1.

We verified the expression of miRNAs that bind to prognostic immune factors in thyroid cancer tissues and paracancerous tissues in the THCAGA database as follows: miR-21-5p, miR-181a-5p, miR-181b-5p, miR-34a-5p, miR-15-5p, miR-182-5p, and miR-181-2-3p were significantly up-regulated tissues. miR-34b-5p, miR-30c-5p, miR-34b-5p, miR-144-5p, miR-30a-3p, miR-204-5p, miR-195-5p, miR-133b, miR-7-2-3p, miR-101-3p, miR-144-3p, miR-218-5p, let-7g-5p, and miR-486-5p were significantly downregulated in tumor tissues. miR-4793, miR-4698, and miR-575 were not significantly changed (**Figures 4A–Y**).

**Figure 5:**
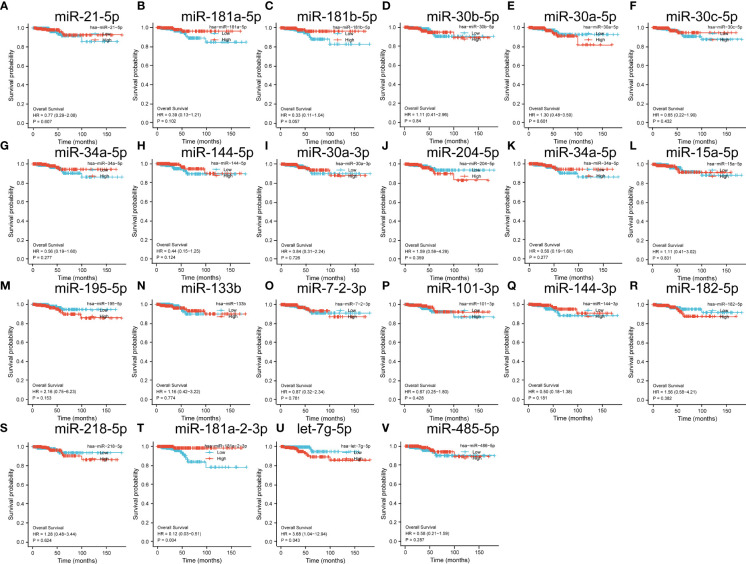
**(A–V)** is a significantly different miRNA in the GEO database that has a target binding relationship with a prognostic factor and the impact on the prognosis of thyroid cancer.

Subsequently, we verified the prognostic impact of the expression profiles of these miRNAs on THCA. The results showed that among these significantly different miRNAs, only miR-181-2-3p significantly impacted the prognosis of THCA (**Figures 5A–V**).

### Correlation between miRNA and prognostic immune factors

**Figure 6:**
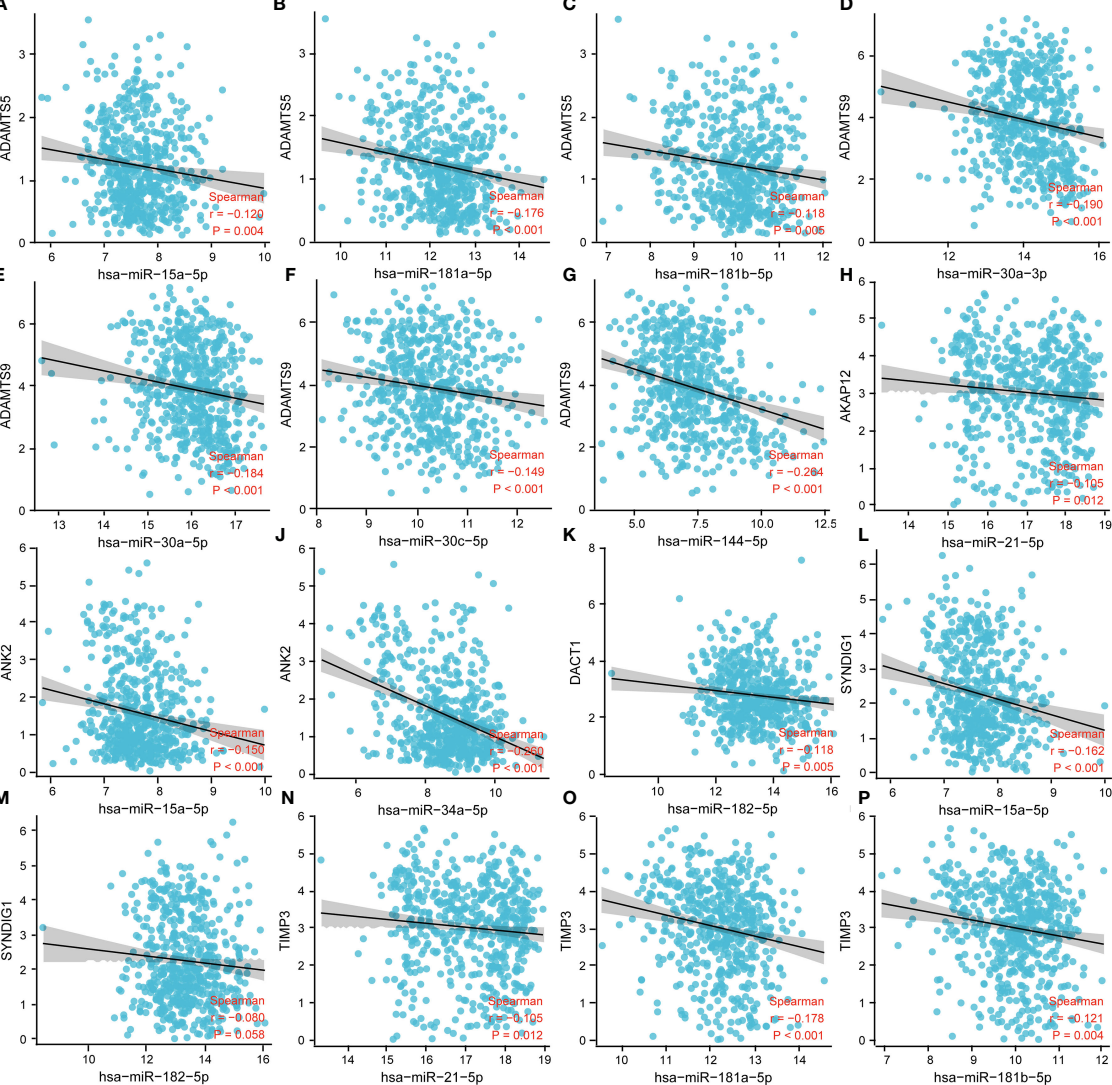
Correlation analysis between miRNA and prognostic immune factors. **(A–C)** *ADMTS5* with miR-15a-5p, miR-101-3p, miR-181a-5p, miR-181b-5p; **(D–G)** *ADMTS*9 with miR-30a-5p, miR-30a-3p, miR-30c-5p, miR-144-5p; **(H)** AKAP12 with miR-21-5p. **(I, J)** *ANK2* with miR-15a-5p, miR-34a-5p; **(K)** *DACT1* with miR-182-5p; **(L, M)** *SYNDIG1* with miR-15a-5p, miR-182-5p; and **(N–P)** *TIMP3* with miR-21-5p, miR-181a-5p, and miR-181b-5p. P < 0.05 was statistically significant.

To further validate the mechanisms of miRNA regulation involved in the expression of prognostic factors, we analyzed the targeted regulatory relationships between miRNAs and prognostic factors. The results are shown in **Figure 6**. The figure shows the following: *ADMTS5* was significantly and negatively correlated with miR-15a-5p, miR-101-3p, miR-181a-5p, and miR-181b-5p. *ADMTS9* was significantly and negatively correlated with miR-30a-5p, miR-30a-3p, miR-30c-5p, and miR-144-5p. *AKAP12* was significantly and negatively correlated with miR-21-5p. *ANK2* was significantly and negatively correlated with miR-15a-5p and miR-34a-5p. *DACT1* was significantly and negatively associated with miR-182-5p. *SYNDIG1* was significantly and negatively related to miR-15a-5p and miR-182-5p. *TIMP3* was significantly and negatively related to miR-21-5p, miR-181a-5p, and miR-181b-5p.

### Correlation of experimental cellular miRNAs with target genes

**Figure 7:**
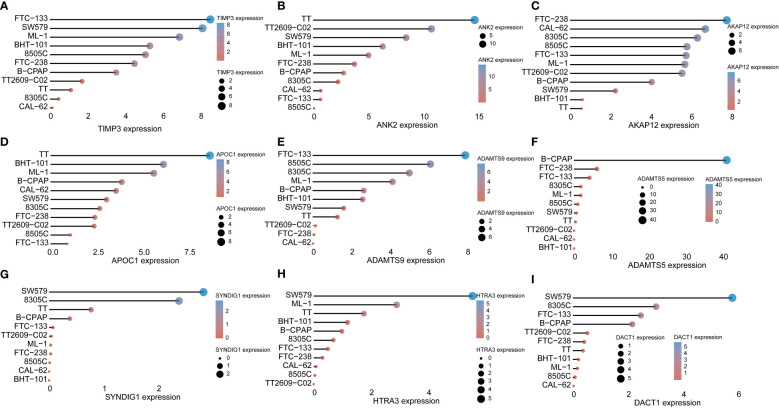
Immune prognostic factors expressed in CCLE data Verification **(A-I)** AKAP12, APOC1, TIMP3, ADAMTS9, ANK2, HTRA3, SYNDIG1, ​​ADAMTS5, and DACT1 Nine immune-related prognostic factors in TT, BHT-101, ML-1, B-CPAP, the expression of 11 thyroid cancer cell lines including CAL-62, SW579, 8305C, FTC-238, TT2609-CO2, 8505C, and FTC-133.

**Figure 8:**
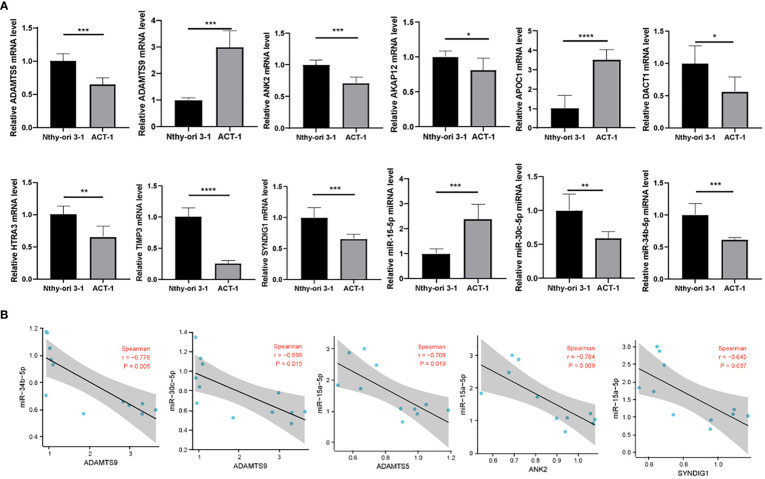
Correlation of experimental cellular miRNAs with target genes. **(A)** RT-qPCR detection of *ADAMTS5, ADAMTS9, ANK2, AKAP12, APOC1, DACT1, HTRA3, TIMP3, SYNDIG1*, miR-15a-5p, miR-30c-5p, and miR-34b-5p expression levels; **(B)** Correlation analysis of miR-15a-5p, miR-30c-5p, and miR-34b-5p and their target genes. * means p <0.05; ** means p < 0.01; *** means p < 0.001; **** means p < 0.0001.

First, we verified the expression of 9 immune-related prognostic factors, AKAP12, APOC1, TIMP3, ADAMTS9, ANK2, HTRA3, SYNDIG1, ​​ADAMTS5 and DACT1, in 11 thyroid cancer cell lines in the CCLE database. Eleven thyroid cancer cell lines expressed to varying degrees (**Figures 7A–I**). We verified the expression of immunoprognostic factors in the cells, as shown in **Figure 8A**. Compared with the normal human thyroid cell line Nthy-ori 3-1, the human THCA cell line ACT-1 showed significant upregulation of *ADAMTS9, APOC1*, and miR-15a-5p. *AKAP12, TIMP3, ANK2, HTRA3, SYNDIG1, ADAMTS5, DACT1*, miR-30c-5p, and miR-34b-5p showed significant down-regulation (**Figure 7A**). We then compared the correlation of miR-15a-5p, miR-30c-5p, and miR-34b-5p with their target genes and found that miR-15a-5p showed a significant negative correlation with *ADAMTS5*, *ANK2*, and S*YNDIG1.* miR-30c-5p and miR-34b-5p showed a significant negative correlation with *ADAMTS9* (**Figure 8B**).

## Discussion

Tumor immunity is associated with proto-oncogene activation and tumor immune escape; therefore, understanding tumor-associated immune genes is crucial for tumor immunity research. In recent years, THCA immune studies have focused primarily on developing immune prognostic models (19–21), and less research has been involved in the regulatory mechanisms of prognostic immune factors. This study mainly analyzed the relationship between immune prognostic factors and miRNAs in THCA. We provided a theoretical basis for further understanding of the immune gene regulation mechanism of THCA and a reference for assessing the prognosis of THCA by analyzing the correlation between the two.

This study analyzed the relationship between immune genes and prognosis using THCAGA and immunoinformatics databases. Twenty-one genes with significant differences in the immune prognosis of THCA were screened, and nine prognostic immune factors were identified by constructing a LASSO risk model (*AKAP12, APOC1, TIMP3, ADAMTS9, ANK2, HTRA3, SYNDIG1, ADAMTS5*, and *DACT1*). This risk model predicted OS after five years with high accuracy (AUC = 0.832).

The nine immune genes in the model have biological characteristics and are related to the clinical prognosis of tumor patients. AKAP12 is an A-kinase scaffold protein with a characteristic binding structural domain of the protein kinase A regulatory subunit. AKAP12 deletion is associated with increased cancer susceptibility (22). *AKAP1* expression is associated with *APOC1* overexpression, promotes tumor progression, and has a poorer prognosis for patient survival (23). *TIMP3* is a metalloproteinase that belongs to the TIMP family. It has a high affinity for proteoglycans in the ECM and its broadest range of substrates, including all MMPs, ADAMs (a disintegrin and metalloproteinase), and ADAMTSs (ADAMs with thrombospondin motifs) (24).

*TIMP-3* has been shown to have anti-metastatic effects by inhibiting matrix metalloproteinases and members of the ADAM family and down-regulating angiogenesis (25). *ADAMTS5* and *ADAMTS9* belong to the ADAMTS (disintegrin-like and metalloproteinase with platelet abasicin motif) proteins. They are extracellular zinc metalloproteinases that play an essential role in extracellular matrix assembly and degradation, connective tissue structure, angiogenesis generation, and cell migration (26). *ADAMTSL5* plays a role in maintaining the function of critical oncogenic signaling pathways, suggesting that it may act as a significant regulator of tumorigenicity and drug resistance (27). adamts9 regulates cancer cell growth and metastasis (28) and is associated with patient survival (29).

*ANK2* has a high mutation frequency in some cancers (30) and silencing of *ANK2* expression reduces the growth and invasion of cancer cell type. *HTRA3* has been implicated as a tumor suppressor in cancer progression in several cancer types, *HTRA3* expression is negatively correlated with adaptive immune cell abundance (T helper cell 17 cells) and positively correlated with innate immune cells (natural killer cells, macrophages, etc.); abundance is positively correlated (31). *SYNDIG1* is a prognostic immune factor in diffuse large B-cell lymphoma and breast cancer (32, 33). *DACT1* belongs to the DACT (Disheveled-associated antagonist of β-catenin) family and is a methylation biomarker for DACT1 in esophageal squamous cell carcinoma (34).

The above data suggest that the nine immune genes in our constructed risk model can exist as prognostic factors in some tumors, reflecting the accuracy of our constructed risk model from the side.

In many studies, the regulatory mechanisms of prognostic factors remain unclear. Based on this starting point, this study analyzed the correlation between immune prognostic factors and miRNAs through TCGA and GEO databases to find the miRNA mechanism of immune prognostic factors in thyroid cancer species.

It has been shown that miRNAs can regulate the target genes involved in the prognosis of tumor patients. For example, in nasopharyngeal carcinoma, the negative correlation between miRNA-19a-3p and *PDCD5* expression levels, miRNA-19a-3p targeting to suppress PDCD5 expression, miRNA-19a-3p levels correlated with N classification and clinical stage of nasopharyngeal carcinoma patients, and PDCD5 levels correlated with T classification, pathological grade, and clinical stage. Survival analysis showed that the expression of high levels of miRNA-19a-3p or low levels of PDCD5 had a poorer prognosis in patients with nasopharyngeal carcinoma (35).

Our study identified 16 groups of miRNAs that showed negative correlations with prognostic immune factors, with specific target-binding relationships confirmed in other cancers. For example, *TIMP3* was downregulated in cervical cancer and was related to poor prognosis in cervical cancer patients, and miR-21-5p target binding to *TIMP3* modulated the development of cervical cancer (36). In gastric cancer, the miR-21-5p binding relationship with *TIMP3* is related to drug resistance in gastric cancer (37). In prostate cancer, the miR-181b-5p-*TIMP3* axis regulates prostate cancer proliferation, migration, and invasive ability (38).

This study also found that by constructing a miRNA regulatory network with certain selected candidate factors (39–42), we could provide a new approach to cancer diagnosis and treatment. The miRNA regulatory network of prognostic immune factors was screened. We also validated at the cellular level that miR-15a-5p showed a significant negative correlation with *ADAMTS5, ANK2*, and *SYNDIG1*. miR-30c-5p and miR-34b-5p showed a significant negative correlation with *ADAMTS9*, but no functional gene. However, no further validation of the functional role of the genes was performed, which will be the direction of our later research.

In summary, nine immunoprognostic factors (AKAP12, APOC1, TIMP3, ADAMTS9, ANK2, HTRA3, SYNDIG1, ADAMTS5, and DACT1) were screened as biomolecular markers for predicting THCA. A prognostic assessment model and risk score system were constructed to predict THCA 5-year OS rates with high and low-risk groups using risk scores. The 5-year OS rate of THCA was predicted with high accuracy. By constructing a prognostic immune factor miRNA regulatory network, we can provide a theoretical basis for the prognostic immune regulatory mechanism of THCA.

## Data availability statement

The original contributions presented in the study are included in the article/supplementary material. Further inquiries can be directed to the corresponding author.

## Author contributions

YT writing the article, writing the revision of article, collect data. TX writing the article, designing the methods, analysis and interpretation. XS writing the article, writing the revision of article, designing the methodology. All authors contributed to the article and approved the submitted version.
