# Erratum: Analysis of the regulatory mechanisms of prognostic immune factors in thyroid cancer

**DOI:** 10.3389/fonc.2023.1281583

**Published:** 2023-09-26

**Authors:** Frontiers Production Office

**Affiliations:** Frontiers Media SA, Lausanne, Switzerland

**Keywords:** thyroid cancer, immune gene, prognosis, miRNA, targeted combination

Due to a production error, there was an error regarding the authorship for **“**Yin Tian” and “Tao Xie”. Both authors should have been listed as co-first authors. The publisher apologizes for this mistake.

The original version of this article has been updated.

